# The Story of Don Miff

**Published:** 1886-05

**Authors:** 


### BIBLIOGRAPHY.

### BOOKS AND PAMPHLETS RECEIVED.

*The story of Don Miff*, as told by his friend John Bouche Whacker. A symphony of life. Edited by Virginius Dabney. New York, J. B. Lippencott & Co., 1886. Price $1.50.

It has been popularly supposed that gentleman belonging to the learned profession of medicine do not read novels.

Now whether this delusion has been nursed by members of that profession with a view towards impressing the laity with the immensity of their practice, or whether the idea was deliberately disseminated by the laity themselves who are never happy if not deluded, must remain for the present an open question. But this is the eventful period of the world's history when the delusion must perforce be laid aside, for though we blush to say it, physicians have recently appeared before the world as the actual perpetrators of novels; and, as it is well known that a long course of assiduous and secret novel reading must have preceded this catastrophy the fact can no longer be concealed. Not only in this degenerate county but in England, and on the continent doctors do read novels, and do enjoy them very much. Such being the case, and acknowledged as such, publishers can no longer refuse to the medical press the romances which they so freely bestow upon the magazines of general literature. This is the first happy result which we hope to observe. The next is, that physicians finding that they can now do openly what they have heretofore practiced in secret, will experience a manifest improvement in their moral character, which though hardly needed, will yet not come amiss.

These thoughts obtrude themselves not unnaturally after the careful perusal of the novel whose name is given at the head of this Review. Of all its good points, which shall we mention? To the Virginian who reads this notice we will say, it is the noble work of a worthy son of Virginia, representing her fair daughters and her brave sons, her old fashioned hospitality and lavish generosity, her happy slaves, and finally her desolated homes! To the humorist we can say, take this book in your hand with confidence, believing that it will afford you many hours of pleasure, though the fun is not unmitigated, and the humor runs often in satire! To the philosopher we would give the assurance that here he will find many chapters much to his taste, but not so much philosophy as to spoil the pure romance which physicians love, for do not romance and sentiment mingle so intimately with our daily toil as to have become almost necessary to a physician's life? Who but he detects the hidden romance in many a home where to all others it is invisible? But this is a theme on which we may not dwell; for are we not writing a Review?

Be advised then our dear doctors to buy this delightful book of Mr. Virginius Dabney. Write to him at his home at 108 West 49th St, New York, and assure him that a good novel is a fitting companion for a good physician. Enclose to him $1.50 lest his publisher should cut him off with one edition, and then, while some misguided brother is idly gossiping in the corner drug store, take you this book "Don Miff" into your sanctum sanctorum and be happy.

